# Sabotage at the Powerhouse? Unraveling the Molecular Target of 2-Isopropylbenzaldehyde Thiosemicarbazone, a Specific Inhibitor of Aflatoxin Biosynthesis and Sclerotia Development in *Aspergillus flavus*, Using Yeast as a Model System

**DOI:** 10.3390/molecules24162971

**Published:** 2019-08-16

**Authors:** Cristina Dallabona, Marianna Pioli, Giorgio Spadola, Nicolò Orsoni, Franco Bisceglie, Tiziana Lodi, Giorgio Pelosi, Francesco Maria Restivo, Francesca Degola

**Affiliations:** Department of Chemistry, Life Sciences and Environmental Sustainability, University of Parma, Parco Area delle Scienze 11/A, 43123 Parma, Italy

**Keywords:** aflatoxin inhibitor, mitochondrion, respiratory chain, yeast model system

## Abstract

Amongst the various approaches to contain aflatoxin contamination of feed and food commodities, the use of inhibitors of fungal growth and/or toxin biosynthesis is showing great promise for the implementation or the replacement of conventional pesticide-based strategies. Several inhibition mechanisms were found taking place at different levels in the biology of the aflatoxin-producing fungal species such as *Aspergillus flavus*: compounds that influence aflatoxin production may block the biosynthetic pathway through the direct control of genes belonging to the aflatoxin gene cluster, or interfere with one or more of the several steps involved in the aflatoxin metabolism upstream. Recent findings pointed to mitochondrial functionality as one of the potential targets of some aflatoxin inhibitors. Additionally, we have recently reported that the effect of a compound belonging to the class of thiosemicarbazones might be related to the energy generation/carbon flow and redox homeostasis control by the fungal cell. Here, we report our investigation about a putative molecular target of the 3-isopropylbenzaldehyde thiosemicarbazone (mHtcum), using the yeast *Saccharomyces cerevisiae* as model system, to demonstrate how the compound can actually interfere with the mitochondrial respiratory chain.

## 1. Introduction

Aflatoxin (AF) contamination of food and feed commodities is a major health concern for sanitary authorities all over the world, since these mycotoxins represent a health hazard to both animals and humans [1]. In particular, aflatoxin B_1_ is considered to be one of the most carcinogenic natural products. *Aspergillus flavus* and *Aspergillus parasiticus* are the main producers of this type of mycotoxin, and both fungi have a wide geographic distribution and may contaminate agricultural crops important for animal and human diet [2]. Moreover, global climate changes have exacerbated the diffusion of these mycotoxigenic *Aspergilli* in regions that, until recently, were considered to be temperate climate areas and were not prone to their growth and their overwinter persistence [3,4]. 

AF contamination may occur pre-harvest or post-harvest owing to fungal infection of crops in the fields or during storage. Accordingly, different approaches have been developed to counteract mycelia and/or mycotoxin contamination. These include good agronomic practices to prevent plant stress that may weaken plant defense or stimulate mycotoxins biosynthesis by the fungus, or chemical treatments to avoid damage of kernels by insects and bio-control by using natural competitors to displace the threatening organism from the ecological niche [3]. Additionally, AF is highly persistent on the various food matrices and is scarcely degraded by the industrial transformation procedures. Carryover of AFs along the food chain may be the primary cause of acute toxicosis in humans and animals, however the possibility that the Aspergillus infection of tissues, organs etc., which is generally referred to as aspergillosis, may lead to AF production “*in situ*” (i.e. in the affected body district) should not be discarded [5,6,7,8,9]. Moreover, mycotoxin production may increase the virulence of aspergillosis by depressing the immunological response of the organism [10]. The availability of specific inhibitors of AF biosynthesis may thus be useful not only in an agronomic perspective, but also as a weapon to counteract the consequences of fungal infection in humans and animals. 

An impressive amount of data is reported in the literature concerning the fungistatic and anti-toxin activity of natural derived and synthetic compounds. However, the molecular mechanisms involved in the specific inhibition of AF synthesis by the relevant compound remain rather speculative in many cases, even if several metabolic/regulatory targets have been suggested [11,12,13,14,15,16,17,18,19,20,21,22,23,24,25,26,27,28,29,30,31]. At present, we are facing a general consensus that AF has a prominent role in the oxidative stress response by the fungus even if, due to the complexity of the regulatory and metabolic steps that are involved in the response, several details of the framework are still unavailable.

We have recently described a set of molecules belonging to the class of thiosemicarbazones, that were tested for their fungicidal and/or inhibitory activity on mycotoxin accumulation on cereal crops [32,33,34]. These molecules, which are very attractive for their coordinating versatility and have the possibility to easily modify the molecular backbone and tune their physical and chemical properties, were designed in order to take advantage of the individual antimycotic properties of metal ions (such as copper, for instance) and natural aldehydes contained in spices are known for their antimycotic properties. For this scope, the aldehydes were functionalized with thiosemicarbazone to endow them with chelating properties towards metal ions. Unexpectedly, these intermediates also showed interesting properties. In particular one of the most promising of them, the thiosemicarbazone of the meta-isomer of cuminaldehyde, namely isopropylbenzaldehyde thiosemicarbazone (mHtcum), displayed a specific activity against aflatoxin biosynthesis distinct from a fungistatic effect, and was therefore investigated in depth to help understand its mode of action in the fungal cell [33]. 

The data gathered by the above-mentioned experiments were consistent with a possible molecular target involved in the energy generation/carbon flow and the redox homeostasis control by the cell: in particular, some evidence suggested that mHtcum could interfere at the mitochondrial level. Here, we describe a set of experiments performed in the yeast *Saccharomyces cerevisiae* addressed to the characterization of mHtcum effect and to the identification of its possible molecular target(s). Even though yeast does not possess the secondary metabolism pathways involved in aflatoxin synthesis, it shares all basal pathways for energy production with other fungi and will be used as a model to corroborate our hypothesis.

## 2. Results and Discussion

### 2.1. Effect of mHtcumon the Oxidative Carbon Source Utilization

**Figure 1:**
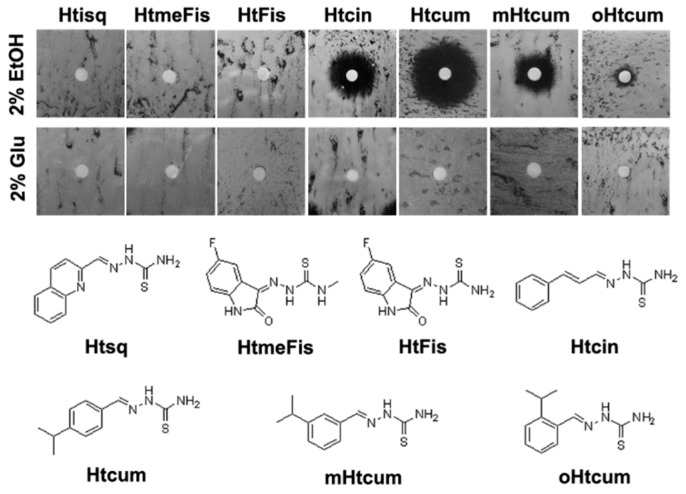
Structure of tested compounds and their effect on yeast oxidative growth. Inhibition halos were evaluated on glucose and ethanol through the agar disk-diffusion method.

On the basis of genetics as well as biochemical and proteomic data, it has been previously speculated that isopropylbenzaldehyde thiosemicarbazone (mHtcum) may negatively affect aflatoxin biosynthesis in *Aspergillus flavus* by redirecting carbon flow in the cell and by modulating the activity of enzymes involved in energy metabolism [33]. In order to investigate whether mHtcum activity may be coupled to a switch from fermentative to respiratory metabolism (or *vice versa*), we first performed a series of experiment using the yeast *S. cerevisiae* as a model system. We analyzed the effect of mHtcum on the utilization of fermentable (glucose) and non-fermentable (ethanol) carbon source by performing a disk diffusion assay, as reported in Figure 1. The effect of other thiosemicarbazones, previously described for their antifungal and antiaflatoxigenic effect [32], was also compared.

On glucose, no growth inhibition was observed for all the compounds. In contrast, in an ethanol-containing medium, an inhibition halo was observed in the case of Htcin, Htcum, mHtcum and oHtcum, i.e. the molecules that inhibit aflatoxin biosynthesis in *A. flavus* at the highest level. Similar results were obtained when ethanol was replaced with other non-fermentable carbon sources such as glycerol and acetate (Figure S1, Supplementary Material), leading us to exclude the possibility of an ethanol-specific effect.

**Figure 2:**
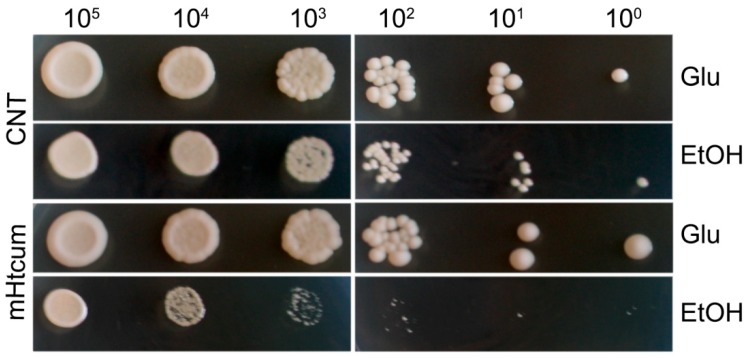
Yeast dilution bioassays showing the effect of mHtcum under oxidative growth. Cells of W303-1B strain serially diluted and spotted on YP medium supplemented with glucose or ethanol and added with mHtcum 50 µM or 0.5% DMSO (CNT).

In addition, the response of *S. cerevisiae* to the mHtcum antiaflatoxigenic concentration of 50 μM during a non-fermentable metabolism was tested with a spot assay (Figure 2); an inhibitory effect of the thiosemicarbazone on yeast cell proliferation was detectable at the 10^4^ cells/spot concentration, and dramatically increased as the cell concentration decreased. 

### 2.2. Interference of mHtcum on Mitochondrial Activity 

The observation that mHtcum negatively affects yeast growth only in the presence of an oxidative carbon source points to a possible molecule-induced mitochondrial impairment. Thus, we wondered if mHtcum might interfere with mitochondrial respiratory-linked processes in *S. cerevisiae*, in particular with the respiratory activity and the mitochondrial cytochromes content, but also with gametogenesis. In fact, nutrient sensing, and in particular the carbon/energy status, plays a critical role in yeast sporulation, and various respiratory inhibitors are reported to affect this developmental process [35,36]

**Figure 3:**
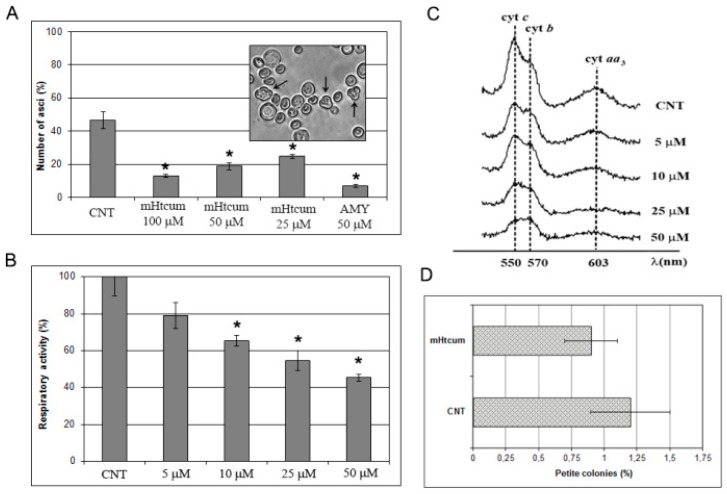
Interference of mHtcum on mitochondrial activity in *S. cerevisiae*. (**A**) Asci production in W303 diploid strain treated with mHtcum. The effect of thiosemicarbazone was compared with the effect of the mitochondrial respiratory inhibitor antimycin A (AMY). Values are expressed as percentage of asci with respect to the number of total cells; statistically significant differences from the control (CNT) were indicated with an asterisk (*p* ≤ 0.05). (**B**) Oxygen consumption rate. W303-1B grown in the absence (CNT) or in the presence of mHtcum at different concentrations (from 5 to 50 µM). Values were normalized to the untreated strain and represented as the mean of at least three values ± SD. Values significantly different from CNT were indicated with an asterisk (*p* ≤ 0.05). (**C**) Reduced versus oxidized cytochrome spectra: peaks at 550, 560 and 602 nm correspond to cytochromes c, b and aa_3_, respectively. The height of each peak relative to the baseline is an index of cytochrome content. (**D**) Mitochondrial DNA mutability. Frequency of respiratory deficient (*petite*) cells determined in untreated (CNT) or 50 μM mHtcum treated yeast cultures, expressed as a percentage.

For the yeast sporification assay, the diploid strain W303, pre-grown for five days in YP medium, was incubated in the sporification medium added with mHtcum 25, 50 and 100 μM. Asci production was estimated after 7 days; antimycin (AMY), a specific inhibitor of the mitochondrial respiratory chain, was used as a reference. As expected, AMY highly affected the number of asci; at the same time, mHtcum was highly effective in lowering yeast sporulation in a dose-dependent manner (Figure 3A).

The effect of mHtcum treatment on oxygen consumption was then measured in yeast cells pre-grown for 18 hours in the presence of different concentrations of mHtcum. As reported in Figure 3B, mHtcum thiosemicarbazone caused a concentration-dependent decrease of respiration, reaching a 55% of inhibition at 50 µM. Accordingly with the drastic impairment of the respiration rate, the profile of mitochondrial cytochromes of yeast cultures was negatively affected by mHtcum treatment in a concentration-dependent manner (Figure 3C). 

The decrease of cell respiration and of the number of cytochromes might be due to the inhibition of at least one of the steps of the electron flow along the respiratory chain by mHtcum. On the other hand, since proteins belonging to the respiratory chain are encoded both by nuclear and mitochondrial genes, the alteration of OXPHOS metabolism in the presence of mHtcum might be due to a mutagenic effect on mitochondrial DNA, with a consequent absence of mitochondrial encoded respiratory proteins. In order to discriminate between these two hypothesis, we measured the frequency of cytoplasmic mitochondrial mutants (*petite* mutants) displaying a respiratory deficient phenotype after treatment with mHtcum. Data reported in Figure 3D show that treatment with thiosemicarbazone of the wild type yeast strain W303-1B did not increase mitochondrial DNA mutability in terms of the *petite* mutants ratio. Results are consistent with our previous data suggesting that mHtcum exerts a sort of rescue effect on *petite* high-frequency yeast mutants [33], owing at least in part to its antioxidant activity.

To evaluate if the long-term effect of mHtcum in yeast cells during growth may be exerted at the level of respiratory complex proteins, the relative abundance of COR1 and COR2 (core subunit 1 and subunit 2 respectively of the ubiquinol-cytochrome c reductase; complex III), COX2 (subunit II of cytochrome c oxidase; complex IV) and ATP2 (beta subunit of the F1 sector of mitochondrial F1F0 ATP synthase; complex V) was estimated in the same treated cultures used for the oxygen consumption rate and discussed above.

**Figure 4:**
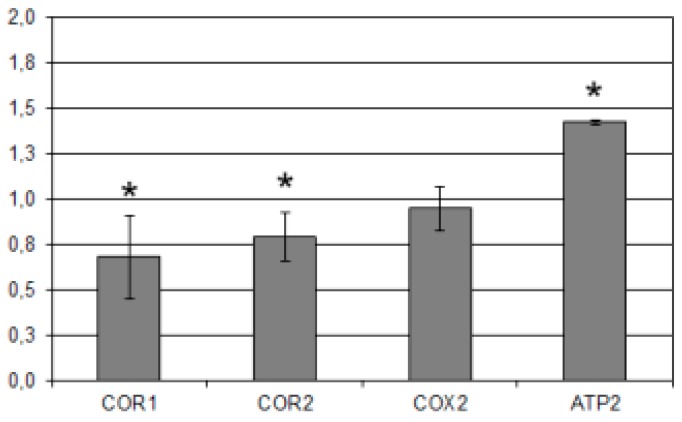
Western Blot analysis of mitochondrial extracts from yeast cells grown in presence of 50 µMm Htcum. Quantification of immunoreactive luminescence signals was achieved by densitometric scanning of the respective bands. Signals were normalized to the 0.5% DMSO treated cultures (control); POR was used as a loading control. Quantification was performed on three independent blots and reported as mean of values (±S.D.). * *p* ≤ 0.05 vs. control.

Densitometric analysis of immunoreactive signals revealed that COR1 and COR2 level was significantly decreased by mHtcum treatment with respect to the control, while the level of COX2 was not significantly affected (Figure 4). Even more remarkably, an increase of ATP2 in yeast cells was detected: the up-regulation of the beta subunit of mitochondrial ATP synthase was in fact observed also in *A. flavus* cultures treated with mHtcum, as described in Degola et al. [33].

**Figure 5:**
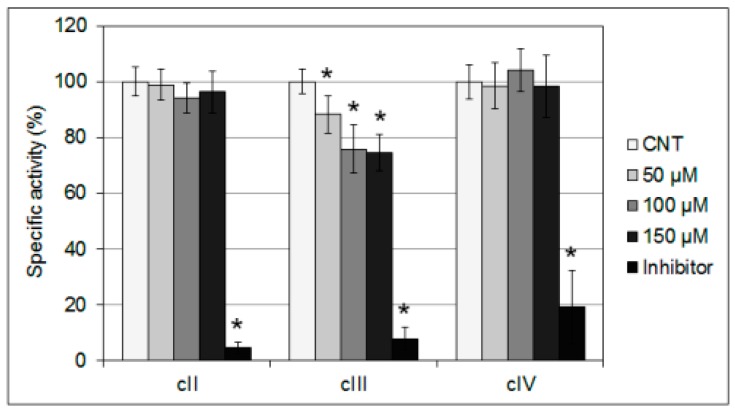
Respiratory chain complexes activity. Biochemical activities of succinate quinone DCPIP reductase (cII), NADH-cytochrome c oxidoreductase (cIII) and cytochrome c oxidase (cIV) were measured on a mitochondrial enriched fraction. mHtcum was added at increasing concentrations (from 50 to 150 µM) and compared to DMSO treated cells (CNT). Specific inhibitors for complex II (malonate), complex III (antimycin A) and complex IV (Na-azide) were used as control. Values were normalized to the control (CTN) and represented as the mean of three independent experiments ± S.D. (* *p* ≤ 0.05).

The effect of the thiosemicarbazone on the enzymatic activity of complexes II, III and IV was then evaluated: an enriched fraction of mitochondria extracted from yeast cultures was used for the spectrophotometric assays, in which the thiosemicarbazone was added at increasing concentrations (from 50 to 150 µM) and compared with the relevant specific inhibitors (malonate, AMY and Na-azide respectively). A negative effect of mHtum was observed on the complex III, whose specific activity decreased by 25–30% at the higher thiosemicarbazone concentrations (Figure 5). In contrast, complex II and IV activities seemed to be insensitive to the addition of mHtcum at any concentration tested (Figure 5). This result supports the hypothesis of a defect in the electron flow along the respiratory chain induced by mHtcum, suggesting, in particular, a specific inhibition of complex III.

### 2.3. Comparative Molecular Docking Analysis

The most studied inhibitor of complex III is Antimycin A, that interacts with the Q_i_/Qn site of cytochrome c reductase in mitochondrial complex III [37], causing a collapse of the proton gradient across the mitochondrial inner membrane, leading to the loss of the membrane potential and, as a consequence, an increase of ROS production and a reduction of the ATP intracellular level [38,39]. We wondered, therefore, if mHtcum inhibition of complex III might be due to its ability to compete with the substrate at Q_i_ site, interfering with the electron flow. Thus, a structure-based docking study was performed to investigate the intermolecular interactions between our thiosemicarbazone and the mitochondrial complex III. Since the inhibitory mechanism of AMY is caused by its binding at bc1cytochrome level in the Q_i_ site, resulting in the disruption of the Q-cycle and the entire enzyme turn over, the docking method was developed using GOLD v5.5 and optimized to compare the theoretical positioning of mHtcum compared to AMY. Structural information about the protein was taken from the Protein Data Bank accession 3BCC (10.2210/pdb3BCC/pdb), antimycin bound to the cytochrome bc1 complex from chicken with AMY, as co-crystallized inhibitor, located in the Q_i_ region. The protein model was prepared by deleting solvent molecules and AMY from the structure and by adding the hydrogen atoms. A 6Å-cavity centered in the Q_i_ region of the protein was used as binding site. A 50-run docking approach was used and the results obtained were scored using the Chemscore fitness function. All the poses were then rescored using the ChemPLP function. The entire docking protocol was validated using the self-docking approach, and AMY was docked successfully [40,41], with RMSD 1.403 Å.

**Figure 6:**
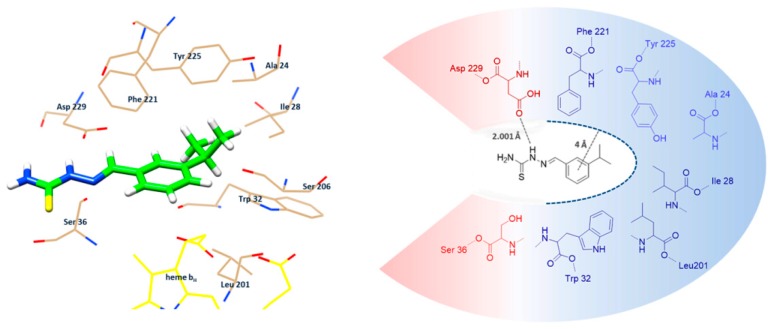
Representation of the binding mode of mHtcum in the cytochrome bc1 AMY binding site as obtained with the Gold v5.5 software. On the left: best docked pose of mHtcum into the 3BCC binding pocket. On the right: schematic positioning of mHtcum into the 3BCC binding pocket (in red: the hydrophilic area; in blue, the hydrophobic surface).

Figure 6 shows the best pose of mHtcum into the Q_i_ site: in detail, the hydrophobic environment formed by Phe^221^, Ile^28^, Tyr^225^, Ala^24^, Trp^32^ and Leu^201^ [42] allows the formation of a binding pocket in which the aromatic ring of mHtcum can be efficiently stabilized. In addition, a strong hydrogen bond (2.001 Å) between thiosemicarbazone N^2^ and the carboxyl group of Asp^229^ is predicted: this bond may convincingly be responsible for the maintenance of mHtcum into the binding site, facing the aromatic portion of the ligand with the hydrophobic cavity of the protein. Noticeably, as for AMY, mHtcum resulted close to heme b_H_ (3.376 Å); this proximity is considered to be fundamental for the inhibition mechanism of AMY, since the b_H_ heme is involved in the oxidation of ubiquinol to ubiquinone, a crucial step in the Q-cycle.

The use of the Chemscore fitness function enables also to predict a binding energy value for each pose [43,44]. This tool was very useful since the energy value obtained from AMY self-docking was used as a reference to evaluate the binding strength of mHtcum in the Q_i_ region. Very similar binding energies were obtained for thiosemicarbazone and AMY, ΔG_bind_ = −5.96 kcal/mol and ΔG_bind_ = −6.05 kcal/mol respectively, suggesting that mHtcum is able to bind at Q_i_ site thus decreasing the activity of mitochondrial complex III.

### 2.4. A. Flavus Growth, Development and Gene Expression 

Recently, Sakuda and coworkers [23] have shown that different respiration inhibitors such as Na-azide, rotenone, oligomycin and antimycin A, that specifically affect different steps of the respiratory chain, inhibit the biosynthesis of aflatoxin in *A. flavus* without significantly interfere with fungal growth. However, no correlation between the inhibitor target along the respiratory chain and the level of inhibition activity on the toxin accumulation have been demonstrated, suggesting that a block of respiration “*tout court*” may be the cause of the inhibition of aflatoxin biosynthesis. Due to the similar behavior observed for mHtcum, we wondered if the compound may mimic the activity of one of the natural inhibitors of respiration reported above. 

**Figure 7:**
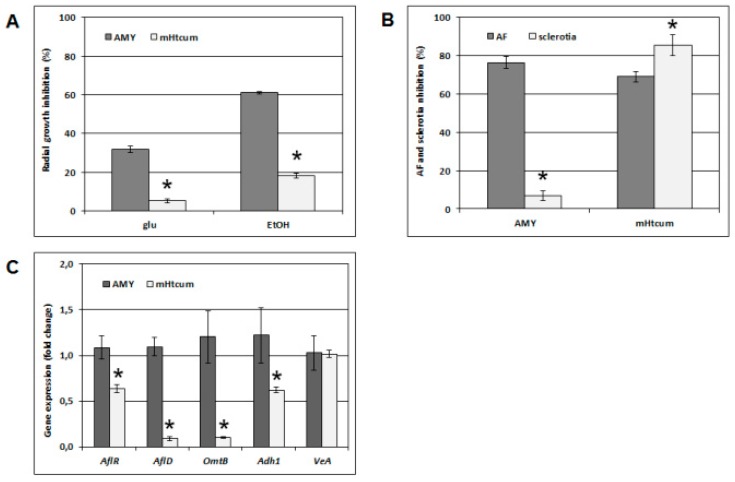
Comparison between mHtcum and antimycin effect on *A. flavus*. (**A**) Differential effect on growth under fermentable/non-fermentable conditions. Fungal growth was assessed as radial growth inhibition of mHtcum or AMY treated *A. flavus* colonies (50 μM) with respect to the control (0.5% DMSO treated cultures). Statistically significant differences respect to AMY treatment are indicated (* *p* < 0.05). (**B**) Effect of mHtcum and AMY on aflatoxin production and sclerotia development. *A. flavus* conidia were inoculated either in CCM or CZ medium for the aflatoxin production and sclerotia development determination respectively (see Materials and Methods section for details). Media were amended with 50 μM mHtcum or AMY, and 0.5% DMSO as control. Statistically significant differences with respect to AMY treatment are indicated (* *p* < 0.05). (**C**) Gene expression fold change of in response to mHtcum and AMY treatment (50 μM). *A. flavus* conidia were incubated and cultivated in the identical cultural conditions used for aflatoxin determination. The 1.0 line represents the control (0.5% DMSO) expression level. Asterisk indicates the differences that were statistically significant (* *p* < 0.05).

All previously discussed evidence seemed to confirm the hypothesis that in yeast cells a common molecular target, or probably one of many, might be shared by mHtcum and AMY, and that this target is located at the mitochondrial level, controlling the functionality of the respiratory chain. We then compared the effect of 50 µM antimycin and mHtcum on *A. flavus*; the fungus radial growth was evaluated on a fermentable (2% glucose containing medium) and a non-fermentable carbon source (2% ethanol containing medium). Similarly to what was observed in sucrose fed cultures, in which the thiosemicarbazone did not significantly affect either conidia germination or mycelium growth [33], the *A. flavus* colonies inhibition rate in glucose was lower than 5% with respect to the control. In contrast, in the presence of a non-fermentable carbon source, mHtcum exposure led to a 18% of radial growth inhibition. Even at a higher level, AMY treated cultures showed the same behavior (Figure 7A). 

The response of *A. flavus* in terms of aflatoxin accumulation and sclerotia biogenesis when treated with AMY or mHtcum was additionally evaluated in order to compare the effect of these molecules on physiological processes belonging to the fungus secondary metabolism. Consistently with Sakuda et al. [23] antimycin A was effective in lowering aflatoxin accumulation at an inhibition level comparable to mHtcum (up to 75% and 70% respectively). In contrast, the sclerotia production was not affected by the presence of AMY, while the *A. flavus* cultures showed to be highly sensitive to the treatment with the thiosemicarbazone (up to 85% of sclerotia inhibition with respect to the control; Figure 7B).

An additional element of the differential activity exerted by the two compounds was then obtained through the analysis of specific gene expression: surprisingly *AflR*, the major regulator of the aflatoxin gene cluster, and the *aflD* and *omtB* genes, encoding for two structural enzymes directly involved in the toxin biosynthesis, proved to be non-down-regulated by antimycin A, contrary to what was observed with the mHtcum treatment (Figure 7C). Similar results were previously reported by Sakuda et al. [23] for inhibitors of mitochondria complex I and II, reinforcing the hypothesis that the mechanism involved in the block of aflatoxin production by antimycin A (and the above mentioned inhibitors) might be only in part shared (differently from the one triggered) by mHtcum. This, in fact, showed down-regulation of the expression of both *aflR* and the other two structural genes (*aflD* and *omtB*) (Figure 7C).

## 3. Materials and Methods

### 3.1. Thiosemicarbazones

Cuminaldehydethiosemicarbazone (Htcum), trans-cinnamaldehyde thiosemicarbazone (Htcin), quinoline-2-carboxyaldehyde thiosemicarbazone (Htisq), 5-fluoroisatin N4-methylthiosemicarbazone (HtmeFis) and 5-fluoroisatin thiosemicarbazone (HtFis) were previously described in Degola et al. [32]; 2-isopropylbenzaldehydethiosemicarbazone (mHtcum) and 3-isopropylbenzaldehyde thiosemicarbazone (oHtcum) were previously described in Degola et al. [33].

### 3.2. Microbial Strains, Media and Culture Conditions

*Aspergillus flavus* strains used in this work were isolated from corn fields of the Po Valley [45] and have already been used for previous experimentation concerning thiosemicarbazone activity [32,33]. *A. flavus* strains are available from the corresponding author on request. Conidia suspensions were obtained from 10-day YES-agar [2% (*w*/*v*) yeast extract (Difco, Detroit, MI), 5% (*w*/*v*) sucrose (Sigma, St Louis, MO), 2% (*w*/*v*) agar (Difco)] cultures incubated at 28 °C; conidia concentration (quantified by OD_600_) and viability (>90%) were determined according to Degola et al. [45]. Coconut milk-derived medium (CCM) used for microplate assays was obtained as described in Degola et al. [46].

*Saccharomyces cerevisiae* yeast strains used in this study were the haploid strain W303-1B (MATα ade2-1 leu2-3,112 ura3-1 trp1-1 his3-11,15 can1-100) [47] and the diploid strain W303 (a/α ade2 leu2 ura3 trp1 his3). All experiments were performed in YP medium (1% yeast extract, 2% peptone, ForMedium). Media were supplemented with various carbon sources as indicated in the text, in liquid phase or after solidification with 20 g/L agar (ForMedium). Growth in the liquid phase was performed with constant shaking at 28 °C. 

### 3.3. Agar Disk-Diffusion Testing

The response of yeast cells to different thiosemicarbazones onto fermentable and non-fermentable carbon sources was assessed in YP medium: W303-1B strain liquid culture was properly diluted to 2.5 × 10^6^ cells/mL, then 400 μL were seeded on YP agar plates supplemented with 2% glucose, 2% ethanol, glycerol or 2% k-acetate alternatively. Filter paper discs (about 6 mm in diameter) were placed on the agar surface, then a volume of 4 μL of 10 mM stock solution of thiosemicarbazones dimethylsulphoxide-resuspended was dropped onto. An equal volume of dimethylsulphoxide (DMSO) was used as control. Petri dishes were incubated at 28 °C, and after 48 h the diameters of inhibition growth zones were evaluated.

### 3.4. Spot Assay for Yeast Oxidative Growth

W303-1B strain were serially diluted from 10^7^ to 10^2^ cells/mL, then 10 μL of each dilution was spotted on YP agar plates supplemented with 2% glucose or 2% ethanol, with or without 50 μM isopropylbenzaldehyde thiosemicarbazone (mHtcum), in order to obtain spots containing from 10^5^ to 10^0^ cell/spot. Plates were incubated at 28 °C.

### 3.5. Asci Production in S. cerevisiae

Diploid W303 yeast strain cells were pre-grown onto 2% glucose YP agar plates for three days at 28 °C. A small portion of culture was then picked up with a sterile loop and straight-inoculated on the surface of SPO IV medium (0.25% (*w*/*v*) yeast extract, 2% (*w*/*v*) K-acetate, 0.1% (*w*/*v*) glucose) agar plates supplemented with thiosemicarbazone mHtcum at increasing concentration (from 25 to 100 μM). DMSO and antimycin A (AMY) were used as negative and positive controls, respectively. Cells were left for sporulation at 28 °C for 6 days, then a small amount of cells was sampled from each treatment, resuspended in bidistilled water, loaded on a slide and observed with an inverted microscope at 200× magnification: three samples from each treatment were observed, and asci from a total of five fields for each samples were counted. Experiments were conducted in triplicate. Results were expressed as the percentage of asci respect with the number of total cells.

### 3.6. Mitochondrial Respiratory Activity and Cytochrome Profiles

Oxygen consumption rate was measured at 30 °C using a Clark-type oxygen electrode (Oxygraph System Hansatech Instruments), with 1 mL of air-saturated respiration buffer (0.1 M phthalate KOH, pH 5.0), 0.5% glucose. Yeast W303-1B cells were cultured at 28 °C in YP medium supplemented with glucose at the non-repressing concentration of 0.6%, until glucose exhaustion, in the presence of isopropylbenzaldehyde thiosemicarbazone (mHtcum) at different concentrations (5, 10, 25, and 50 µM). The control was treated with the same volume of the solvent DMSO. Oxygen consumption was normalized to the dry weight of the cells. Cytochrome profiles were determined spectrophotometrically at room temperature (Varian Cary300 UV-VIS Spectrophotometer), by recording reduced oxidized cytochrome spectra of yeast W303-1B cells cultured for oxygen consumption rate determination. 

### 3.7. Mitochondrial DNA Stability 

For *petite* frequency determination the W303-1B strain was pre-grown at 28 °C in YP medium, supplemented with 2% ethanol in order to counterselect the *petite* cells that were already present in the population. Cells were then inoculated in YP medium supplemented with 2% glucose and isopropylbenzaldehyde thiosemicarbazone at the final concentration of 50 µM, whereas to the control treatment the same volume of DMSO was added; cultures were then incubated at 28 °C. After 15 generations, cells were plated at a dilution which provided approximately 200 cells/plate on SC (0.19% YNB without aminoacids and NH_4_SO_4_ powder ForMedium, 0.5% NH_4_SO_4_, supplemented with 1g/L dropout mix [48]) agar plates containing 2% ethanol and 0.25% glucose. *Petite* frequency was defined as the percentage of colonies showing the *petite* phenotype after a 6-day incubation at 28 °C.

### 3.8. Isolation of Mitochondria, Gel Electrophoresis and Western Blot Analysis 

Mitochondrial proteins for Western Blot analyses were obtained by suspending the cells in extraction buffer containing 0.6 Msorbitol, 10 mM imidazole, 0.5 mM EDTA, 0.1% BSA and 1 mM PMSF. Cells were broken by vortexing on ice using glass beads and mitochondrial proteins were obtained by centrifugation and re-suspended in the extraction buffer. Quantification of protein concentration was performed by Bradford’s method [49], using Bio-Rad protein assay following the manufacturer’s instructions. For each sample, 15 µg of mitochondrial total proteins were loaded on 12% polyacrylamide gel, subjected to SDS-PAGE, and Western Blot was performed. Gels were electroblotted onto nitrocellulose filters and successively immunostained with specific antibodies against CORE1 and CORE2 (kindly provided by Prof. B.L. Trumpower), COX2 (Molecular Probes, Invitrogen), and ATP2 (kindly provided by Prof. J.P. di Rago). POR1 (Abcam Mitoscience, Cambridge, UK) was used as the loading control. After incubation with the appropriate secondary antibodies, ECL Western Blotting Substrate (Clarity^TM^, BioRad, Hercules, CA, USA) was used for final detection. Signals were quantified through QuantityOne Software (Bio-Rad, Hercules, CA, USA).

Enzymatic activities: Complex II (succinate dehydrogenase), complex III (cytochrome c reductase) and complex IV (cytochrome c oxidase) specific activities were measured spectrophotometrically according to Barrientos et al. [50] on a mitochondrial-enriched fraction prepared as previously described [51]. Thiosemicarbazone mHtcum was added to the cuvette. As control the same volume of DMSO was added. 

### 3.9. Aflatoxin Production Assay

Effect on aflatoxin biosynthesis was assessed by the microplate fluorescence-based procedure described in Degola et al. [46]. Standard flat-bottom 96-well microplates (Sarstedt, Newton, NC, USA) were used. Suspensions of conidia were diluted and brought to the final concentration of 5 × 10^2^ conidia/well; cultures were set in a final volume of 200 µL/well of CCM medium, added of the test molecule at 50 μM. DMSO 0.5% (*v*/*v*) was used as the control. The plates were incubated in the dark under stationary conditions for 6 days at 25 °C. Aflatoxin accumulation was monitored directly in the culture medium by fluorescence emission determination using a TECAN SpectraFluor Plus microplate reader (Männedorf, Switzerland; λ_ex_ = 360 nm; λ_em_ = 465 nm; manual gain = 83; lag time = 0 µs; number of flashes = 3; integration time = 200 µs). Samples were inoculated in quadruplicate.

### 3.10. A. flavus Radial Growth Determination

*A. flavus* radial growth on a fermentable and a non-fermentable carbon source was performed in YES-agar medium supplemented with 2% glucose (*w*/*v*)and 2% ethanol (*v*/*v*) respectively, and amended with mHtcum or antimycin A at the concentration of 50 µM. Three single spots (5 µL of a 10^7^ conidia/mL suspension each) of the aflatoxigenic strain were equidistantly inoculated in Petri dishes (9 cm Ø), while plates were reviewed in triplicate and incubated for 4 days at 25 °C, and the mycelium growth was evaluated daily by measuring colonies reverse along two orthogonal diameters. The radial growth rate was expressed as percentage inhibition with respect to the control (±S.D.).

### 3.11. Sclerotia Biogenesis Assay

A volume of 5 μL from a 10^5^ spores/mL suspension of *A. flavus* aflatoxigenic strain was point inoculated centrally on Czapek Agar plates [1.5% (*w*/*v*) agar, 0.1% (*w*/*v*) di-potassium hydrogenphosphate, 0.05% (*w*/*v*) magnesium sulfate heptahydrate, 0.05% (*w*/*v*) potassium chloride, 0.3% (*w*/*v*) sodium nitrate,3% (*w*/*v*) sucrose] added with mHtcum or antimycin A at the concentration of 50 μM. Plates were incubated up to 10 days to obtain sclerotia production; then, the surface of colonies was scratched, and sclerotia were manually recovered, ethanol-washed to remove conidia, dried up to 3 days at 60 °C and weighted. Data were recorded as dry weight (mg) and converted into sclerotia production percentage inhibition with respect to the control (±S.D.). Plates were inoculated in four replicates.

### 3.12. RNA Extraction and Gene Expression Analysis

Total RNA was extracted from 200 mg of mycelium sampled from 96-h-old microplate CCM cultures treated with mHtcum and antimycin A 50 µM, using a TRIzol® Kit (Sigma-Aldrich, Saint Louis, MO, USA) according to the manufacturer’s instructions. Reverse-transcription of 2 µg of total RNA was conducted using the Maxima First-Strand cDNA Synthesis Kit for qRT-PCR with dsDNase (Thermo Fisher Scientific, Waltham, MA, USA), following the manufacturer instructions. The complementary DNA samples were used as templates of qPCR reactions conducted with ABI 7300 instrumentation (Thermo Fisher Scientific, Waltham, MA, USA) and iTaq™ Universal SYBR® Green Supermix (BioRad, Hercules, CA, USA). Primer sequences, already described in Degola et al. [33], are reported in Table 1. Target genes expression was normalized to the expression of *tub1* gene. The ΔCT was calculated as CT target gene −CT internal standard; the expression level variations were then expressed as 2^−ΔΔCT^ (with ^ΔΔ^CT = CT treatment − CT control). Three biological and three technical replicates per condition were performed. Amplification conditions were: 50 °C for 2 min and 95 °C for 10min; 40 cycles at 95 °C for 15 s and 60 °C for 1 min; 95 °C for 15 s, 60 °C for 1 min, 95 °C for 15 s, and 60 °C for 15 s. The significance of differences between relative expression ratios of treated and control samples was determined using the Mann-Whitney test (*p* ≤ 0.05).

### 3.13. Statistical Analysis

For statistical analyses, one-way analysis of variance (ANOVA) was used in the Past 3.x software. Results of mycelial growth, aflatoxin accumulation, and sclerotia production were analyzed by Tukey’s test; differences were considered significant at *p* ≤ 0.05.

## 4. Conclusions

Fungi secondary metabolism and, in particular, mycotoxins biosynthetic pathways require a well-defined regulation of oxidative intracellular balance triggering the redox homeostasis needed for both fungal development and differentiation [52]. Because of this pivotal role, destabilization of oxidative-linked systems, such as mitochondrial electron flow, can be an effective way to control the fungal production of aflatoxins. The data presented in this paper suggest that the toxin inhibition induced by mHtcum is linked to molecular switches regulating the carbon source utilization and the energy production in the cell. The interaction between the compound and bc1 cytochrome in the active site of mitochondrial complex III, proposed in consideration of the results obtained in the yeast model system, has been supported by a docking analysis validation. On the other hand, since in *A. flavus* aflatoxin biosynthesis and sclerotia biogenesis, that are both coordinately inhibited by mHtcum treatment, do not equally respond to a specific inhibitor of complex III, the existence of multiple (at least two) targets for thiosemicarbazone should be presumed. Thus, we propose that mHtcum might operate on a regulatory step that is located upstream of the bifurcation of aflatoxin biosynthesis and sclerotia development pathways. However the overall regulatory machinery might be more sophisticated since it should be considered that, as a mycelia forming fungus, *Aspergillus* could go through differentiation/specialization processes, resulting in a task division (some cells engage in energy metabolism, whereas other do so in the secondary metabolites production).

## Figures and Tables

**Table 1:** Oligonucleotides sequence and target genes used in this study.

Oligo Name	Sequence (5′3′)	Target Gene
tubFw-RT	TACCATGGACGCCGTCCG	*Tub1*
tubRev-RT	GACGGACAACATCGACAAC	
OmtB F	GCCTTGACATGGAAACCATC	*AflO*
OmtBRev-RT	TCCACTGCTCAATCGCATG	
Adh1 FwRT	CTAAACCAGGACCAGATGAG	*Adh1*
Adh1 RevRT	TCCCTCGTGTCCACCTAC	
Nor1 F	ACGGATCACTTAGCCAGCAC	*AflD*
AflD-RevRT	ACGGTGCTTTTGGGACGTTG	
AflJ-gF	GAACGCTGATTGCCAATGCC	*AflS*
AflS RevRT	GATTCATCCAGAGGGATAC	
VeA FwRT	CGAGACGGAAGCCTCCGT	*VeA*
VeA RevRT	TGGAGGATCGACTGGACGA	

## Supplementary Materials

The following are available online. Figure S1: Effect of mHtcum on yeast cells growth on different non-fermentable carbon sources. In the disc diffusion assay, ethanol was replaced by acetate or glycerol as carbon source in the YP medium.
