# Supplementary figures and images for: Sabotage at the Powerhouse? Unraveling the Molecular Target of 2-Isopropylbenzaldehyde Thiosemicarbazone, a Specific Inhibitor of Aflatoxin Biosynthesis and Sclerotia Development in Aspergillus flavus, Using Yeast as a Model System
Source: Molecules. 2019 Aug 16;24(16):2971. doi: 10.3390/molecules24162971 (PMC6719062; doi:10.3390/molecules24162971)

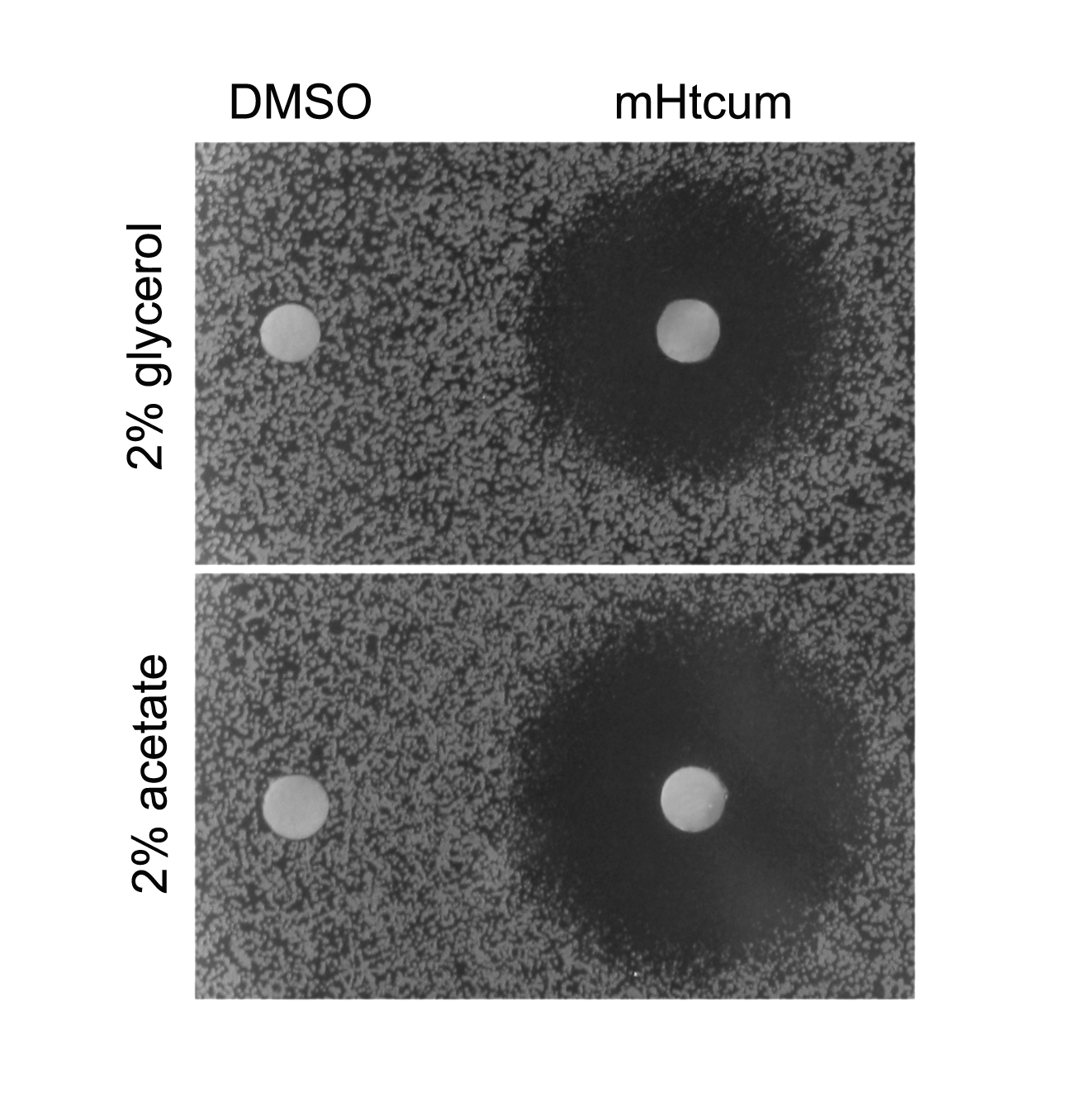

Supplement: Supplementary file 1 [file molecules-24-02971-s001.zip › molecules-558154-supplementary.tif]
